# Comparison between two- and three-dimensional methods for offset measurements after total hip arthroplasty

**DOI:** 10.1038/s41598-022-16952-3

**Published:** 2022-07-25

**Authors:** Shine Tone, Masahiro Hasegawa, Yohei Naito, Hiroki Wakabayashi, Akihiro Sudo

**Affiliations:** Department of Orthopaedic Surgery, Mie University Graduate School of Medicine, 2-174 Edobashi, Tsu, Mie 514-8507 Japan

**Keywords:** Outcomes research, Bone, Orthopaedics

## Abstract

The aim of this study was to compare acetabular offset, femoral offset, and global offset measurements obtained after total hip arthroplasty (THA) between a two-dimensional (2D) method and a three-dimensional (3D) method. The subjects were 89 patients with unilateral osteoarthritis who underwent primary THA at our institution. Acetabular, femoral, and global offsets were measured by each of the 2D and 3D methods in native and implanted hips. In native hips, mean acetabular, femoral, and global offsets were 32.4 ± 3.3, 32.7 ± 4.5, 65.1 ± 5.7 mm, respectively, by the 2D method, and 32.3 ± 3.1, 38.1 ± 4.0, 70.4 ± 4.9 mm, respectively, by the 3D method. In implanted hips, mean acetabular, femoral, and global offsets were 27.6 ± 4.1, 33.8 ± 7.8, 61.4 ± 8.5 mm, respectively, by the 2D method, and 27.6 ± 3.9, 41.8 ± 6.2, 69.4 ± 7.2 mm, respectively, by the 3D method. There was significant difference in femoral and global offsets between the 2D and 3D methods in both native and implanted hips. Comparison of the 2D and 3D methods for evaluation of acetabular, femoral, and global offsets after THA clarified the usefulness and accuracy of the 3D method.

## Introduction

Total hip arthroplasty (THA) is a highly successful surgical intervention performed to relieve pain and improve function in individuals with advanced arthritis of the hip joint^1^. A primary goal of THA is to provide a stable hip by restoring joint biomechanics^2^. Reconstruction of joint offsets promotes joint stability, which reduces the risk of dislocation and enables a good range of motion with low risk of bony or soft-tissue impingement, sufficient abductor muscle strength without alteration in gait, and minimized polyethylene wear^2–8^. Clement et al. have reported that the long-held biomechanical theory of medialization of the acetabular component with compensatory increased femoral offset results in improved functional outcome^9^.

Some reports have evaluated offset after THA using a two-dimensional (2D) method based on postoperative anteroposterior radiographs^3,10–12^. However, it has also been reported that offset measurements obtained from radiographs can be underestimated compared with those obtained by computed tomodensitometry and a low-dose EOS imaging system^13,14^ A recent study reported that three-dimensional (3D) preoperative planning based on computed tomography (CT) imaging showed high accuracy and consistency for hip reconstruction^15^. 3D planning software has also been used for the postoperative evaluation of THA^16–18^. To the best of our knowledge, there are few reports to have undertaken a detailed comparison of offset values between the 2D method using postoperative radiographs and 3D bone models obtained from CT data. We hypothesized that the 3D method might be more accurate than the 2D method for evaluating offsets.

The aim of this study was to measure acetabular, femoral, and global offsets after THA, using a 2D method based on postoperative anteroposterior radiographs and a 3D method based on bone models from CT data, and to compare offset values between the two methods. Offset values were also compared between native hips and implanted hips after THA.

## Materials and methods

### Patient selection

**Figure 1:**
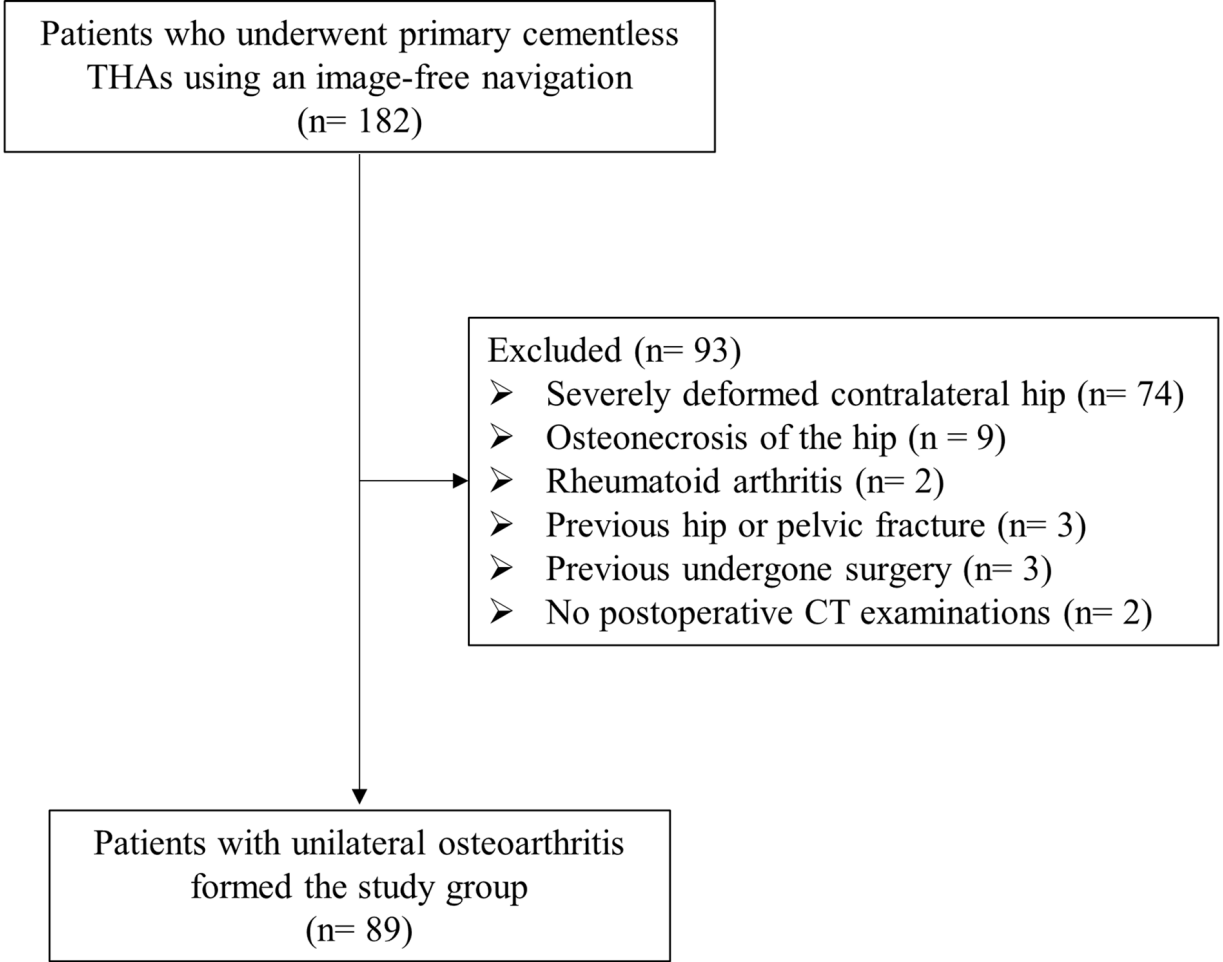
Patient flow diagram.

We performed primary THA with a cementless cup and non-modular cementless stem (Kyocera, Kyoto, Japan) in 191 hips of 182 patients using an image-free navigation system (Brain Lab; KICK Hip application 6.0, Helmstetten, Germany) at our institution between June 2014 and March 2020. Of these, 89/182 patients with unilateral osteoarthritis formed the study group (17 men, 72 women; mean age, 71.7 ± 7.7 years; mean weight, 55.1 ± 11.1 kg; mean height, 152.2 ± 7.9 cm; mean body mass index, 23.7 ± 3.9 kg/m^2^). The diagnosis was secondary osteoarthritis due to hip dysplasia in 73/89 hips (Crowe Group 1 in 62 hips, Group 2 in 9 hips, Group 3 in 2 hips), and primary osteoarthritis in 16/89 hips. The exclusion criteria were severely deformed contralateral hip, osteonecrosis of the hip, rheumatoid arthritis, previous hip or pelvic fracture, previous undergone surgery, and no postoperative CT examinations (Fig. 1). Operations were performed so that the offset of the implanted hip is equal to that of the native hip because included patients have unilateral osteoarthritis.

### Radiological analysis

**Figure 2:**
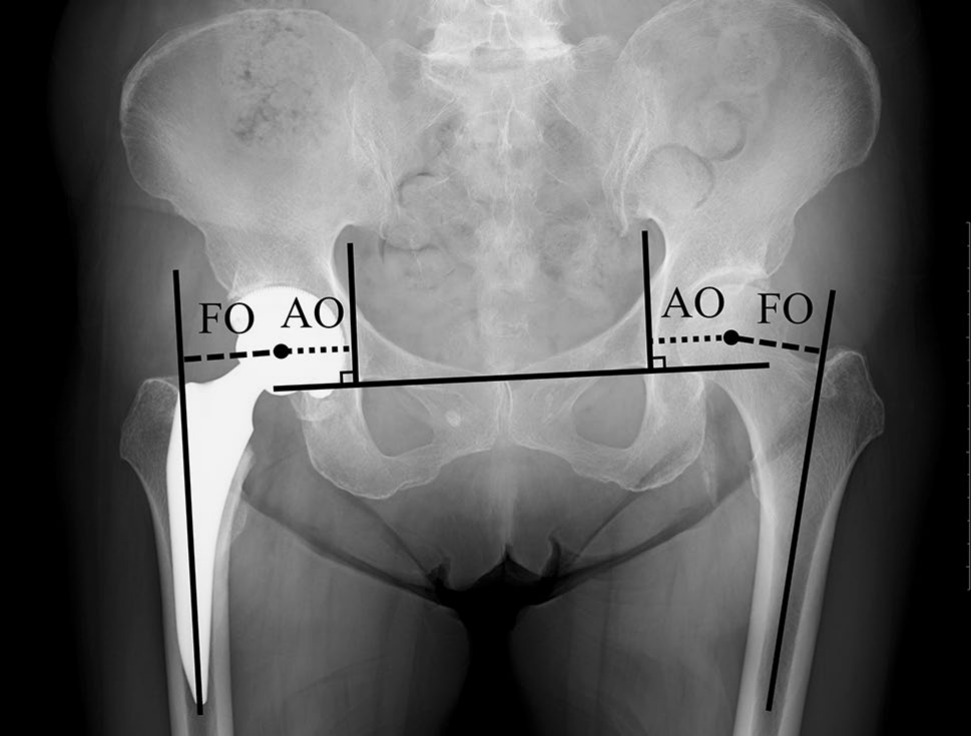
Assessment of acetabular offset (AO) and femoral offset (FO) using the 2D method.

All patients admitted to the study underwent postoperative radiographic and CT examinations for the assessment of offset within 2 weeks postoperatively. Standard anteroposterior pelvic radiographs were obtained with the patient supine, with the lower limbs placed in internal rotation and the big toes touching each other to centre the patella. The 2D method for evaluating offsets was described by Flecher as follows^19^. Acetabular offset was defined as the perpendicular distance from the centre of rotation of the femoral head to the vertical trans-teardrop line. Femoral offset was defined as the distance from the centre of rotation of the femoral head to the central axis of the femur (Fig. 2). Global offset was defined as the sum of the femoral and acetabular offsets.

**Figure 3:**
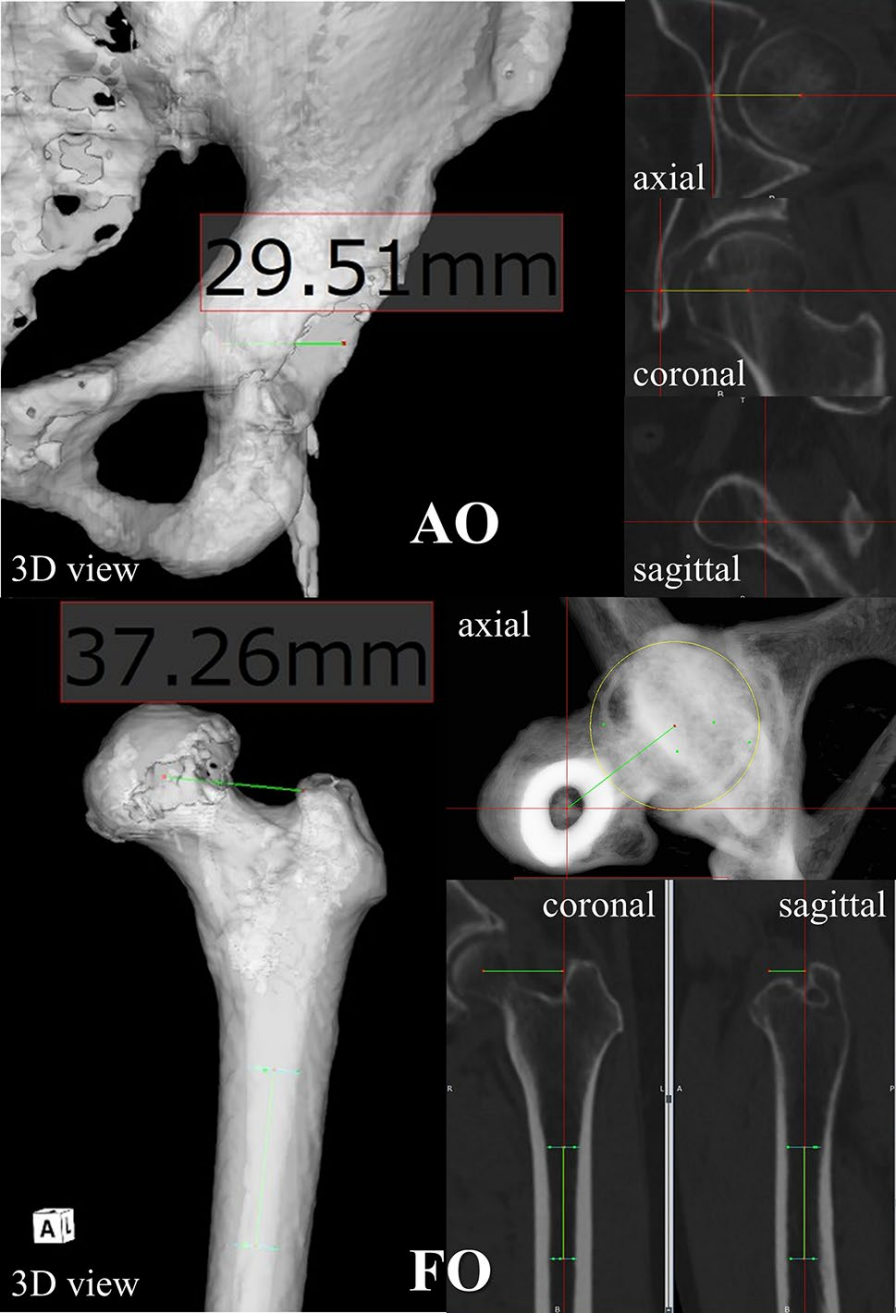
Assessment of acetabular offset (AO) and femoral offset (FO) using the 3D method.

Helical CT with 1-mm slice interval was performed from the anterior superior iliac spine to the knee in all patients. Offset was evaluated by the 3D method using the 3D-template system (ZedHip; LEXI Co., Ltd., Tokyo, Japan) as follows. Acetabular offset for the 3D method was defined as the perpendicular distance from the centre of rotation of the femoral head to the teardrop line in the functional pelvic plane after repositioning using the 3D-template system. Femoral offset for the 3D method was defined as the perpendicular distance from the centre of rotation of the femoral head to a line passing through the centre of two circles created in the proximal medullary cavity of the femur (Fig. 3). In addition, we checked that the line passing through the centre of two circles passed the centre of the femoral proximal medullary cavity by reconstructing the coordinate plane. Global offset for the 3D method was defined as the sum of the femoral and acetabular offsets for the 3D method.

Acetabular, femoral and global offset values in native and implanted hips were compared between the 2D and 3D methods. Offset values were compared between native and implanted hips, and offset error in native and implanted hips was compared between the 2D and 3D methods.

### Statistical analysis

All statistical analyses were performed with EZR (Saitama Medical Centre, Jichi Medical University, Saitama, Japan), a graphical user interface for R (The R Foundation for Statistical Computing, Vienna, Austria)^20^. Normal distribution of values was assessed by the Kolmogorov–Smirnov normality test for each series of measurements. For data with normal distribution, paired Student’s t-test was used for analysis. For data without normal distribution, continuous data were analyzed using the nonparametric Wilcoxon signed-rank test and Spearman’s rank correlation coefficient. Values of *P* < 0.05 were considered significant.

The reproducibility of the 2D and 3D methods was evaluated. For intra-observer reliability, one orthopaedic surgeon measured each parameter twice in 10 hips, at an interval of ≥ 4 weeks. To test inter-observer reliability, two orthopaedic surgeons measured each parameter twice on 10 hips, at an interval of ≥ 4 weeks. Intra-class and inter-class correlation coefficients were calculated to analyse the variability between observers. Values of 0.81–1.00 indicated almost perfect correlation; 0.61–0.80, substantial correlation; 0.41–0.60, moderate correlation; 0.21–0.40, fair correlation; and 0.00–0.20, slight correlation^21^.

### Ethics approval and consent to participate

This study was approved by Institutional Review Board at Mie University hospital (H2018-083). All patients gave informed consent for the use of their surgical data in the study. The study was carried out in accordance with the principles of the Helsinki declaration.

## Results

Table 1 lists the intra-class and inter-class correlation coefficients in native and implanted hips. There was almost perfect correlation for all measurements.

**Table 1:** Intra-class and inter-class correlation coefficients.

	Intraclass correlation coefficient				Interclass correlation coefficient			
	2D		3D		2D		3D	
	Native hip	Implanted hip	Native hip	Implanted hip	Native hip	Implanted hip	Native hip	Implanted hip
Acetabular offset	0.98 (0.94 ~ 1.00)	0.99 (0.95 ~ 1.00)	0.99 ( 0.97 ~ 1.00)	1.00 (0.99 ~ 1.00)	0.97 (0.87 ~ 0.99)	0.97 (0.88 ~ 0.99)	0.97 ( 0.87 ~ 0.99)	0.98 (0.92 ~ 1.00)
Femoral offset	0.92 (0.69 ~ 0.98)	0.98 (0.91 ~ 0.99)	0.93 (0.74 ~ 0.98)	0.97 (0.89 ~ 0.99)	0.94 (0.76 ~ 0.99)	0.99 (0.94 ~ 1.00)	0.91 (0.64 ~ 0.98)	0.95 (0.79 ~ 0.99)
Global offset	0.97 (0.88 ~ 0.99)	0.97 (0.90 ~ 0.99)	0.96 (0.85 ~ 0.99)	0.98 (0.94 ~ 1.00)	0.98 (0.90 ~ 0.99)	0.99 (0.97 ~ 1.00)	0.97 (0.87 ~ 0.99)	0.97 (0.86 ~ 0.99)

In native hips, mean acetabular, femoral, and global offset values by the 2D method were 32.4 ± 3.3, 32.7 ± 4.5, and 65.1 ± 5.7 mm, respectively, and by the 3D method were 32.3 ± 3.1, 38.1 ± 4.0, and 70.4 ± 4.9 mm, respectively.

In implanted hips, mean acetabular, femoral, and global offset values by the 2D method were 27.6 ± 4.1, 33.8 ± 7.8, and 61.4 ± 8.5 mm, respectively; and by the 3D method were 27.6 ± 3.9, 41.8 ± 6.2, and 69.4 ± 7.2 mm, respectively. Statistical analysis revealed no significant difference between the 2D and 3D methods for acetabular offset in either native or implanted hips. However, a significant difference was found for femoral and global offset between the 2D and 3D methods for both native and implanted hips (Table 2). With both the 2D and 3D methods, acetabular offset in implanted hips was significantly smaller than that in native hips. There was no significant difference in femoral offset between native and implanted hips by the 2D method; however, there was significant difference in femoral offset between native and implanted hips by the 3D method. Regarding global offset, no significant difference was observed between native and implanted hips by the 3D method, and global offset was significantly smaller in implanted hips than in native hips by the 2D method (Table 3).

**Table 2:** Comparison between 2 and 3D methods for acetabular offset, femoral offset, and global offset.

	Native hip			Implanted hip		
	2D	3D	*p*	2D	3D	*p*
Acetabular offset (mm)	32.4 ± 3.3 (27 ~ 46)	32.3 ± 3.1 (26 ~ 44)	0.614	27.6 ± 4.1 (20 ~ 40)	27.6 ± 3.9 (19 ~ 41)	0.655
Femoral offset (mm)	32.7 ± 4.5 (21 ~ 41)	38.1 ± 4.0 (27 ~ 47)	< 0.01	33.8 ± 7.8 (16 ~ 54)	41.8 ± 6.2 (29 ~ 58)	< 0.01
Global offset (mm)	65.1 ± 5.7 (53 ~ 78)	70.4 ± 4.9 (58 ~ 84)	< 0.01	61.4 ± 8.5 (44 ~ 85)	69.4 ± 7.2 (56 ~ 91)	< 0.01

**Table 3:** Comparison between native and implanted hips for acetabular offset, femoral offset, and global offset.

	2D			3D		
	Native hip	Implanted hip	*p*	Native hip	Implanted hip	*p*
Acetabular offset (mm)	32.4 ± 3.3 (27 ~ 46)	27.6 ± 4.1 (20 ~ 40)	< 0.01	32.3 ± 3.1 (26 ~ 44)	27.6 ± 3.9 (19 ~ 41)	< 0.01
Femoral offset (mm)	32.7 ± 4.5 (21 ~ 41)	33.8 ± 7.8 (16 ~ 54)	0.186	38.1 ± 4.0 (27 ~ 47)	41.8 ± 6.2 (29 ~ 58)	< 0.01
Global offset (mm)	65.1 ± 5.7 (53 ~ 78)	61.4 ± 8.5 (44 ~ 85)	< 0.01	70.4 ± 4.9 (58 ~ 84)	69.4 ± 7.2 (56 ~ 91)	0.086

**Figure 4:**
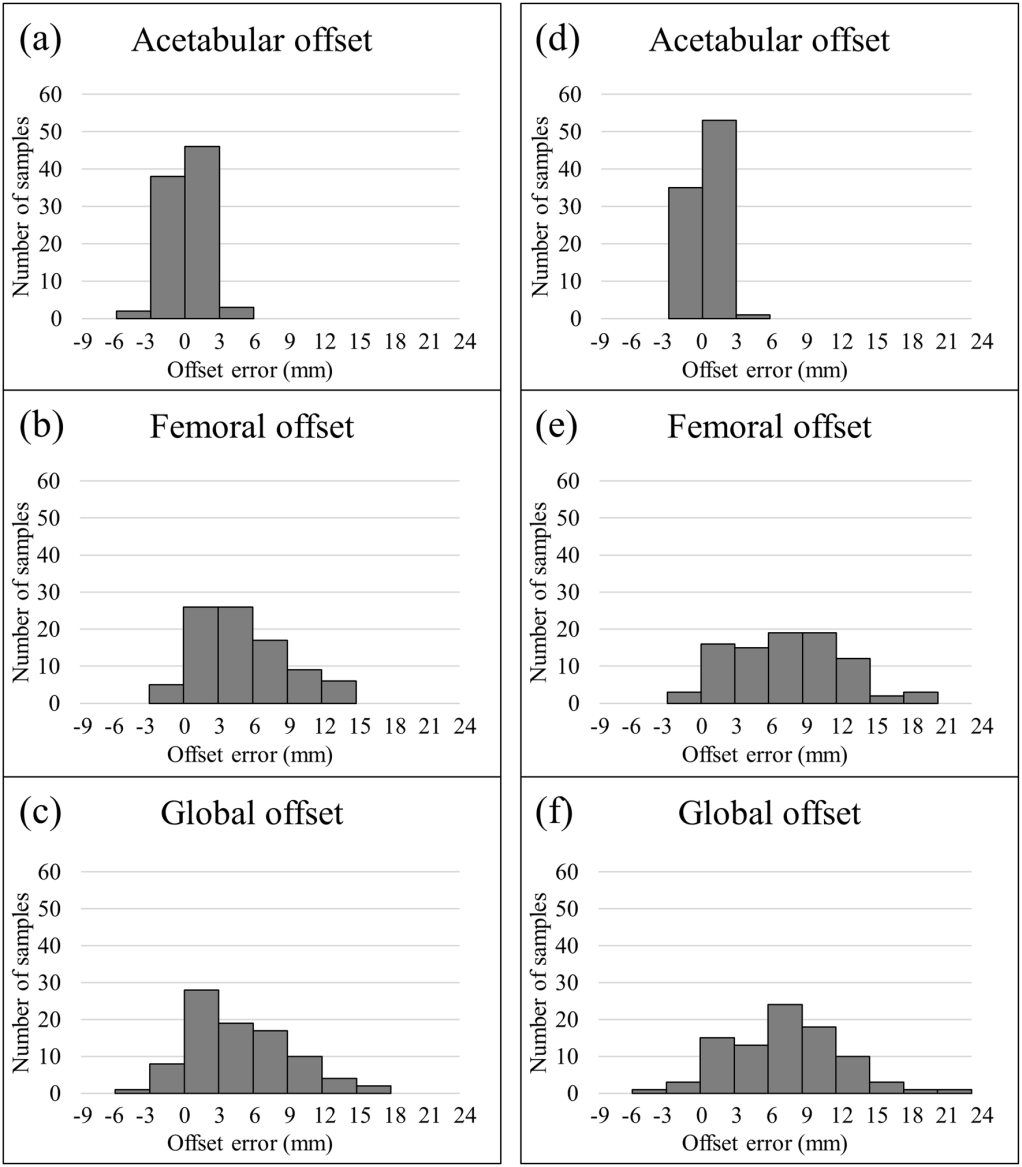
Histogram of offset error between 2 and 3D methods by: (**a**)–(**c**) native hip; (**d**)–(**f**) implanted hip.

**Figure 5:**
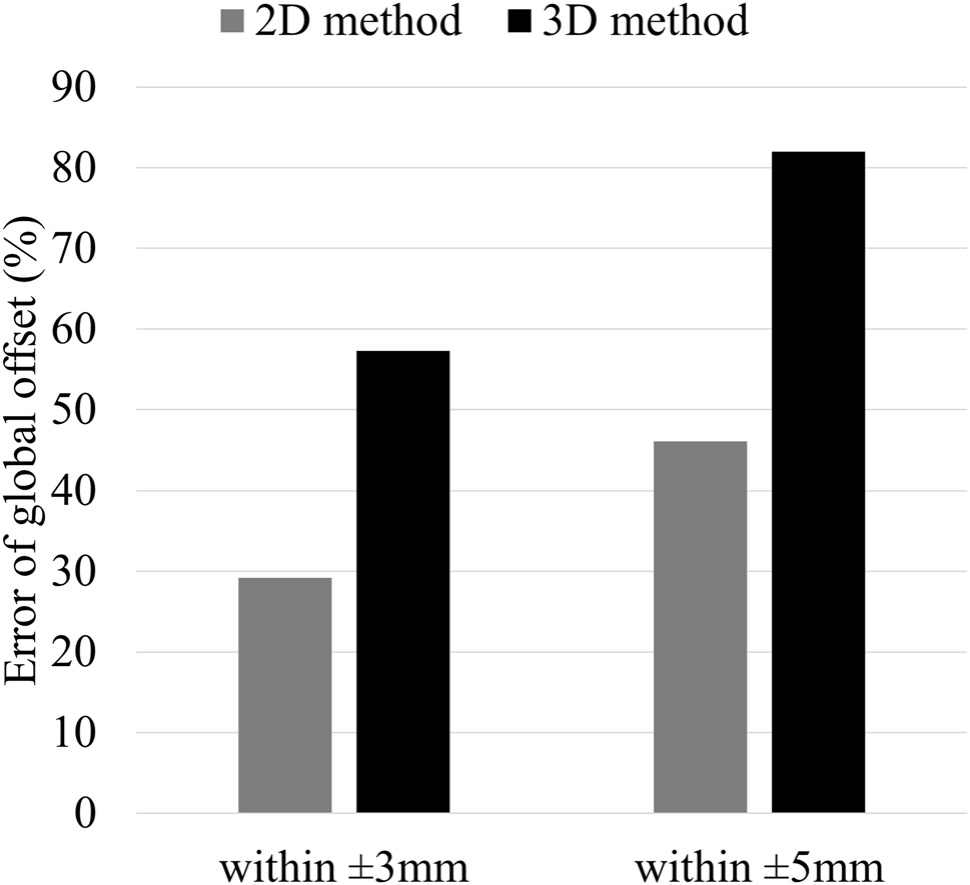
Comparison of 2D and 3D methods for error of global offset between native and implanted hips.

Figure 4 shows a histogram of offset error between the 2D and 3D methods. For acetabular offset, values in all patients were within 6 mm for both native and implanted hips. However, there was considerable variation in femoral and global offset values with errors of up to 24 mm. For error of global offset between native and implanted hips by the 2D method, agreement within ± 3 mm was confirmed in 26/89 THAs (29.2%), and agreement within ± 5 mm was confirmed in 41/89 THAs (46.1%). In contrast, for error of global offset between native and implanted hips by the 3D method, agreement within ± 3 mm was confirmed in 51/89 THAs (57.3%), and agreement within ± 5 mm was confirmed in 73/89 THAs (82.0%) (Fig. 5).

## Discussion

In this study, we measured offset values after THA and compared these measurements between 2 and 3D methods. The main finding was that error between the 2D and 3D methods varied among the offset parameters. There was no significant difference in acetabular offset between the two methods. This result is very important because it indicates that the 2D method can be used to evaluate acetabular offset with the same accuracy as the 3D method. No previous studies have compared acetabular offset between 2 and 3D methods. In addition, postoperative acetabular offset in implanted hips was medialized by 4.8 mm compared to that in the native hip by both the 2D and 3D methods. This result suggests that secondary osteoarthritis such as acetabular dysplasia is common in Japan. Jingushi et al. have reported a 9% incidence of primary osteoarthritis and a 81% incidence of secondary osteoarthritis with hip dysplasia in Japan^22^. Of the present patients, 82% had secondary osteoarthritis of the hip associated with acetabular dysplasia, which makes it difficult to achieve placement in the original anatomical position in THA. To obtain sufficient acetabular cup coverage and favorable clinical outcomes in THA for patients with hip dysplasia, we usually place the cup more medially, then more proximally, and eventually use superolateral bone grafting^19,23,24^. The resultant medialization of the hip centre has been estimated as 3–6 mm^23,25,26^. However, if the decreased acetabular offset was not compensate and only the original femoral offset was restored, the global offset will be reduced. Several studies have reported that change of the hip centre affect the biomechanics of the hip^27,28^. To maintain global offset, medialization should theoretically be compensated for by an equivalent increase in femoral offset^10^. If the surgeon compensates for this decrease in acetabular offset by increasing femoral offset by the same amount this both restores global offset and the abductor lever arm. There are some potential advantages of medialization of the hip centre and maintaining global offset. Bicanic et al. have reported that hip load decreases when the cup is placed more medially or distally, and when the femoral neck is longer or lateral offset is used^29^. Bonnin et al. have also reported that medialization of the cup decreased stresses on the head–cup interface and on the abductor muscles, even when global offset was not restored. Moreover, they found that optimal outcome in terms of stress was observed when the cup was medialised but global offset was restored^30^. Therefore, medialization of the hip centre and maintaining global offset have the positive effect of decreasing hip load and also decreases the risk of bone or soft tissue impingement, thus improving the range of motion of the hip joint and reducing the risk of dislocation^31,32^.

In contrast, some studies have reported underestimation of femoral offset by the 2D method compared with the 3D method. Lazennec et al. compared femoral offset between measurements obtained from radiographs and the EOS imaging system^14^. Femoral offsets for native and implanted hips were 40 and 41 mm, respectively, using radiographs; and 43 and 45 mm, respectively, with the EOS imaging system. Pasquier et al. have reported preoperative and postoperative femoral offset values of 38.97 and 41.83 mm, respectively, for radiographic measurement; and 42.9 and 44.68 mm, respectively, for CT-scan measurement^13^. In addition, Lecerf et al. have reported mean femoral offset of 42.6 mm with radiographic measurement and 45.8 mm with CT-scan measurement^33^. In the present study, femoral offset was significantly underestimated by the 2D method compared with the 3D method in both native and implanted hips. Although femoral offset can be generally measured on anteroposterior radiographs of the femoral neck with the lower limb in about 20 degrees of internal rotation, the reproducibility of this method is poor, having mean error of about 9.7 mm^19^. In addition, internal rotation of 15–20 degrees does not consistently compensate for femoral anteversion, which varies widely even in primary osteoarthritis^4,34^. These results indicate that the 3D method should be used to evaluate femoral offset.

Global offset was also significantly smaller with the 2D method than with the 3D method in both native and implanted hips because femoral offset contributes to underestimation of global offset. Furthermore, the error in global offset between the native and implanted hips was larger with the 2D method than with the 3D method. In their comparison of radiographic and CT-scan measurements, Lecerf et al. have reported that radiographic measurement underestimated by > 5 mm in 28% of cases^33^. In a cadaveric study, Weber et al. have reported that 15% of the radiographic global offset change measurements and 35% of the femoral offset change measurements on plain radiographs were outside a tolerance limit of 5 mm compared to the 3D-CT^35^. In the present study, error in global offset showed considerable variation according to evaluation method. These results indicate that it is difficult to accurately evaluate global offset using the 2D method; therefore, we recommend the 3D method for this measurement.

There are three limitations of the present study. First, it is difficult to plot accurately the centre of rotation when the femoral head is severely deformed. However, the native hip is an almost sphere because of normal hip in this study. In addition, the centre of the head by 3D method can be calculated in three-dimensions by plotting four points around the circumference of the head to create a sphere approximated the head. Second, no deformity of the hip does not necessarily mean that native hip is a normal hip. However, we consider that it has little influence on the results of this study because this study does not assess whether the contralateral hip is normal or not. Third, CT after THA has a higher radiation burden to patients compared with that from radiographic measurements^36^. However, we consider that the benefit of this method outweighs the disadvantage of the higher radiation dose because our results clarified that offset evaluation is more accurate with the 3D method. Furthermore, 3D method for evaluation of offset parameters are not affected by limb position and antetorsion of the femoral head. In the future, we plan to examine the relevance to clinical outcomes using the offset parameters that can be accurately measured by 3D method.

In conclusion, the results of this study clarified differences in accuracy between 2 and 3D methods for evaluation of offset parameters. Although there was no significant difference between the methods for acetabular offset, the 2D method significantly underestimated femoral and global offsets compared with the 3D method. These results demonstrate the accuracy and potential of the 3D method for evaluation of offset parameters following total hip arthroplasty.

## Data Availability

The datasets used and/or analysed during the current study available from the corresponding author on reasonable request.

## References

[1] Ferguson RJ (2018). Hip replacement. Lancet.

[2] Forde B (2018). Restoring femoral offset is the most important technical factor in preventing total hip arthroplasty dislocation. J. Orthop..

[3] Mahmood SS, Mukka SS, Crnalic S, Wretenberg P, Sayed-Noor AS (2016). Association between changes in global femoral offset after total hip arthroplasty and function, quality of life, and abductor muscle strength: A prospective cohort study of 222 patients. Acta Orthop..

[4] Sariali E, Klouche S, Mouttet A, Pascal-Moussellard H (2014). The effect of femoral offset modification on gait after total hip arthroplasty. Acta Orthop..

[5] McGrory BJ, Morrey BF, Cahalan TD, An KN, Cabanela ME (1995). Effect of femoral offset on range of motion and abductor muscle strength after total hip arthroplasty. J. Bone Joint Surg. Br..

[6] Asayama I, Chamnongkich S, Simpson KJ, Kinsey TL, Mahoney OM (2005). Reconstructed hip joint position and abductor muscle strength after total hip arthroplasty. J. Arthroplasty..

[7] Little NJ, Busch CA, Gallagher JA, Rorabeck CH, Bourne RB (2009). Acetabular polyethylene wear and acetabular inclination and femoral offset. Clin. Orthop. Relat. Res..

[8] Sakalkale DP, Sharkey PF, Eng K, Hozack WJ, Rothman RH (2001). Effect of femoral component offset on polyethylene wear in total hip arthroplasty. Clin. Orthop. Relat. Res..

[9] Clement ND, Patrick-Patel RS, MacDonald D, Breusch SJ (2016). Total hip replacement: Increasing femoral offset improves functional outcome. Arch. Orthop. Trauma Surg..

[10] Dastane M, Dorr LD, Tarwala R, Wan Z (2011). Hip offset in total hip arthroplasty: quantitative measurement with navigation. Clin. Orthop. Relat. Res..

[11] Ellapparadja P, Mahajan V, Deakin AH, Deep K (2015). Reproduction of hip offset and leg length in navigated total hip arthroplasty: How accurate are we?. J. Arthroplasty..

[12] Enke O, Levy YD, Bruce WJ (2020). Accuracy of leg length and femoral offset restoration after total hip arthroplasty with the utilisation of an intraoperative calibration gauge. Hip Int..

[13] Pasquier G (2010). Total hip arthroplasty offset measurement: is C T scan the most accurate option?. Orthop. Traumatol. Surg. Res..

[14] Lazennec JY, Brusson A, Dominique F, Rousseau MA, Pour AE (2015). Offset and anteversion reconstruction after cemented and uncemented total hip arthroplasty: an evaluation with the low-dose EOS system comparing two- and three-dimensional imaging. Int. Orthop..

[15] Sariali E, Mouttet A, Pasquier G, Durante E, Catone Y (2009). Accuracy of reconstruction of the hip using computerised three-dimensional pre-operative planning and a cementless modular neck. J. Bone Joint Surg. Br..

[16] Sariali E, Boukhelifa N, Catonne Y, Pascal Moussellard H (2016). Comparison of three-dimensional planning-assisted and conventional acetabular cup positioning in total hip arthroplasty: A randomized controlled trial. J. Bone Joint Surg. Am..

[17] Bayraktar V (2017). Accuracy of measuring acetabular cup position after total hip arthroplasty: Comparison between a radiographic planning software and three-dimensional computed tomography. Int. Orthop..

[18] Inoue D (2015). Value of computed tomography-based three-dimensional surgical preoperative planning software in total hip arthroplasty with developmental dysplasia of the hip. J. Orthop. Sci..

[19] Flecher X, Ollivier M, Argenson JN (2016). Lower limb length and offset in total hip arthroplasty. Orthop. Traumatol. Surg. Res..

[20] Kanda Y (2013). Investigation of the freely available easy-to-use software 'EZR' for medical statistics. Bone Marrow Transplant..

[21] Landis JR, Koch GG (1977). The measurement of observer agreement for categorical data. Biometrics.

[22] Jingushi S (2010). Multiinstitutional epidemiological study regarding osteoarthritis of the hip in Japan. J. Orthop. Sci..

[23] Sanchez-Sotelo J, Berry DJ, Trousdale RT, Cabanela ME (2002). Surgical treatment of developmental dysplasia of the hip in adults: II Arthroplasty options. J. Am. Acad. Orthop. Surg..

[24] Takao M (2011). The results of a press-fit-only technique for acetabular fixation in hip dysplasia. J. Arthroplasty..

[25] Eggli S, Pisan M, Müller ME (1998). The value of preoperative planning for total hip arthroplasty. J. Bone Joint Surg. Br..

[26] Knight JL, Atwater RD (1992). Preoperative planning for total hip arthroplasty Quantitating its utility and precision. J. Arthroplasty..

[27] Shen X, Tian H, Li Y, Zuo J, Gao Z, Xiao J (2022). Acetabular revision arthroplasty based on 3-dimensional reconstruction technology using jumbo cups. Front. Bioeng. Biotechnol..

[28] Hu Y (2022). Postoperative hip center position associated with the range of internal rotation and extension during gait in hip dysplasia patients after total hip arthroplasty. Front Bioeng. Biotechnol..

[29] Bicanic G, Delimar D, Delimar M, Pecina M (2009). Influence of the acetabular cup position on hip load during arthroplasty in hip dysplasia. Int. Orthop..

[30] Bonnin MP, Archbold PH, Basiglini L, Selmi TA, Beverland DE (2011). Should the acetabular cup be medialised in total hip arthroplasty. Hip Int..

[31] Archbold HA (2006). The transverse acetabular ligament: an aid to orientation of the acetabular component during primary total hip replacement: A preliminary study of 1000 cases investigating postoperative stability. J. Bone Joint Surg. Br..

[32] Kurtz WB, Ecker TM, Reichmann WM, Murphy SB (2010). Factors affecting bony impingement in hip arthroplasty. J. Arthroplasty..

[33] Lecerf G (2009). Femoral offset: anatomical concept, definition, assessment, implications for preoperative templating and hip arthroplasty. Orthop. Traumatol. Surg. Res..

[34] Argenson JN (2005). Three-dimensional anatomy of the hip in osteoarthritis after developmental dysplasia. J. Bone Joint Surg. Br..

[35] Weber M (2014). Plain radiographs fail to reflect femoral offset in total hip arthroplasty. J. Arthroplasty..

[36] Brenner DJ, Hall EJ (2007). Computed tomography–an increasing source of radiation exposure. N. Engl. J. Med..

